# 3D Printing of Polymeric Bioresorbable Stents: A Strategy to Improve Both Cellular Compatibility and Mechanical Properties

**DOI:** 10.3390/polym14061099

**Published:** 2022-03-09

**Authors:** Ana M. Sousa, Ana M. Amaro, Ana P. Piedade

**Affiliations:** Department of Mechanical Engineering, CEMMPRE, University of Coimbra, 3030-788 Coimbra, Portugal; amicaelabsousa96@gmail.com (A.M.S.); ana.amaro@dem.uc.pt (A.M.A.)

**Keywords:** vascular stents, polymers, degradation, mechanical properties, 3D printing

## Abstract

One of the leading causes of death is cardiovascular disease, and the most common cardiovascular disease is coronary artery disease. Percutaneous coronary intervention and vascular stents have emerged as a solution to treat coronary artery disease. Nowadays, several types of vascular stents share the same purpose: to reduce the percentage of restenosis, thrombosis, and neointimal hyperplasia and supply mechanical support to the blood vessels. Despite the numerous efforts to create an ideal stent, there is no coronary stent that simultaneously presents the appropriate cellular compatibility and mechanical properties to avoid stent collapse and failure. One of the emerging approaches to solve these problems is improving the mechanical performance of polymeric bioresorbable stents produced through additive manufacturing. Although there have been numerous studies in this field, normalized control parameters for 3D-printed polymeric vascular stents fabrication are absent. The present paper aims to present an overview of the current types of stents and the main polymeric materials used to fabricate the bioresorbable vascular stents. Furthermore, a detailed description of the printing parameters’ influence on the mechanical performance and degradation profile of polymeric bioresorbable stents is presented.

## 1. Introduction

According to the World Health Organization (WHO), cardiovascular diseases are among the most prevalent and leading causes of death worldwide [1]. The WHO estimates that about 17 million deaths annually are related to cardiovascular diseases, and this number will increase up to around 23.6 million by 2030 [2]. Usually, cardiovascular-related illness is associated with disorders in the heart, blood vessels, or both. Several risks are mentioned in literature as a cause of cardiovascular diseases: unhealthy diet, physical inactivity, obesity, hypertension, diabetes, tobacco, and harmful alcohol use [3,4].

**Figure 1:**
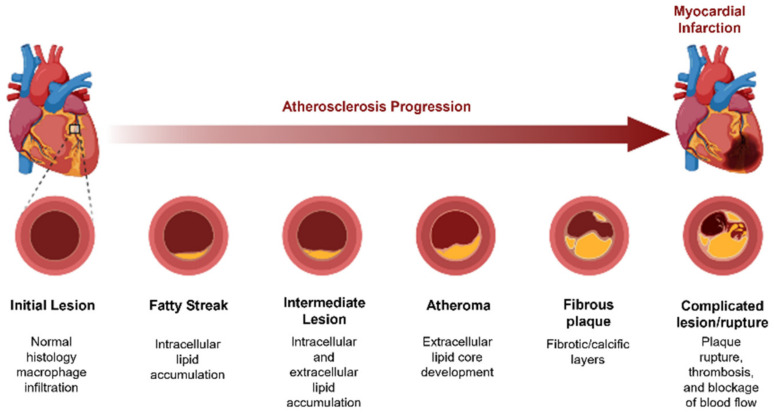
Coronary artery disease: scheme of atherosclerosis progression leading to myocardial infarction (Created in BioRender.com).

Among all cardiovascular problems, the most common is coronary artery disease, also called ischemic heart disease or coronary heart disease. Generally, these problems are related to disorders caused by narrowed heart arteries that supply blood to the cardiac muscle [5]. Coronary artery disease results from the accumulation of plaques in the inner surface of the arteries, a condition medically known as atherosclerosis (Figure 1). These plaques begin to be constituted by cholesterol, fat, and, later, calcifications due to the accumulation of calcium. After some time, atherosclerotic plaques harden, and the arteries become more narrowed (stenosis), limiting the oxygen-rich blood flow in the arterial system. When the arteries that oxygenate the heart are completely blocked, ischemia of the heart will occur, and myocardial infarction or heart attack will be experienced by the patient [5,6].

The first positive effort to treat atherosclerosis emerged with balloon angioplasty, a minimally invasive procedure with minimum costs [6]. This medical procedure involves introducing a guiding catheter, with a balloon, inside the artery that is narrowed, inflating the balloon to reopen the artery and restore the blood flow [7]. However, this procedure also presents some limitations, such as vessel occlusion, which led to the development of vascular stents [8,9]. At the moment, vascular stenting or percutaneous coronary intervention (PCI) is the central therapy used to reopen narrow arteries through stent implantation [10]. Since the development and use of the first stent in 1986, the universal value of the stents market has grown to around USD 7 billion, and it is estimated that it will grow more than 5% annually [11].

Despite the benefits of stenting technology, some mechanical and biological problems still need to be addressed because around 30–40% of patients still experience in-stent restenosis (ISR) after the stent implantation [12]. Thus, it is necessary to optimize and find new approaches to overcome these limitations by employing new manufacturing techniques or materials. The current overview presents the main polymeric materials used to fabricate the bioresorbable vascular stents. Additionally, considering the actuality of additive manufacturing (AM) processes, commonly designated as 3D printing, how the processing parameters can influence the mechanical performance and degradation profile of polymeric bioresorbable stents is discussed.

## 2. Vascular Stenting

Vascular or coronary stents are hollow and tubular structures inserted in an obstructed artery to open and prevent the blockage of the vascular lumen, supporting the vessel wall at the same time [13]. This section reports a wide range of information concerning the current types of available stents and their required properties/characteristics.

### 2.1. Requirements for an Ideal Stent

For all types of stents available in the market, these invasive medical devices must fulfill some requirements to avoid failure during their use. The stent specifications must consider biological, chemical, physical, and mechanical properties [14]. The Food and Drug Administration (FDA) established guidelines and suggestions for stent manufacturing and enumerated certain clinical and relevant features. Although there is no specific design for stents, these devices must fulfill and combine the requirements given by the FDA, some of which are presented in Table 1 [15].

One of the most crucial characteristics of stents is their cellular compatibility aiming to avoid adverse biological responses. Therefore, the device must have a nontoxic and compatible base material to prevent events such as ISR, in-stent thrombosis (IST), and/or neointima hyperplasia in a stented blood vessel [17]. Although many base materials for stents were a revolution in the surgery field, the devices trigger adverse biological events due to their permanent structure. In order to avoid these events, some devices have been made with bioresorbable materials that can support the artery wall during the healing and, after a specific time, be reabsorbed by the organism.

Regarding the mechanical properties, radial strength is the most important to consider during stent fabrication [18,19,20]. Radial strength is defined as “the radial force that the stent can withstand before collapsing”, which means that the device must have enough radial strength to support the forces exerted by the artery wall (radial pressure) [21]. Another important property to consider is the elastic recoil, defined as the reduction in the stent diameter after implantation. Recoil must be diminished to allow the tissue to heal and prevent the narrowing of the blood vessel. If there is a high percentage of elastic recoil, several adverse consequences can occur: stent restenosis, blow disruption, and, in the worst situation, the device moving to another location [6]. In addition, radial strength strongly influences the elastic recoil as the higher the radial strength, the lower the probability of stent recoil. The FDA does not recommend any specific standard to evaluate the radial strength, but several studies have followed the guideline of ASTM F3067-14 [22,23]. Other studies use the tensile test to assess the radial strength and stiffness by relating them directly with other material properties such as ultimate tensile strength and Young modulus, respectively [24]. A study from Al-Mangour et al. [25] states that a high tensile strength helps accomplish a sufficient radial strength to support the artery wall and maintain the lumen area.

The flexibility of vascular stents also should be considered before and after stent implantation. Before implantation, the vascular stent must have enough flexibility to pass inside the blood vessels during delivery, maintaining the shape and the design. Once placed in the artery, the flexibility of the device must be taken into consideration since the vascular stent must have the capacity to bend to be well fitted to the blood vessel [26]. Additionally, it should resist compression from the arteries during the systolic and diastolic movements [23].

It is necessary to evaluate the fatigue properties to assess the long-term durability of cardiovascular stents and their clinical success. Fatigue can lead to the failure of a stent, resulting in the loss of radial support and triggering biological events [27]. Usually, the fatigue of this type of device is influenced mainly by the cyclic loading generated by the heart beating (systolic and diastolic movements). The inner diameter of the artery changes due to the pressure caused by the pulsatile blood flow. Consequently, the stent is exposed simultaneously to bending, torsion, and compression [28]. These events can lead to crack initiation due to the oscillating stress condition in the stent and, therefore, stent fracture [29].

An invasive coronary device can be either a balloon-expandable stent (BE) or a self-expandable stent (SE) considering the deployment method. The most common is the BE procedure, which expands the stent with a balloon catheter, deforming the device plastically. In the SE procedure, the stents have a greater diameter than the artery, and the device is constrained before being positioned. Then, the SE device is released to expand if the base material responds to an external stimulus, for instance, temperature [27,28].

**Figure 2:**
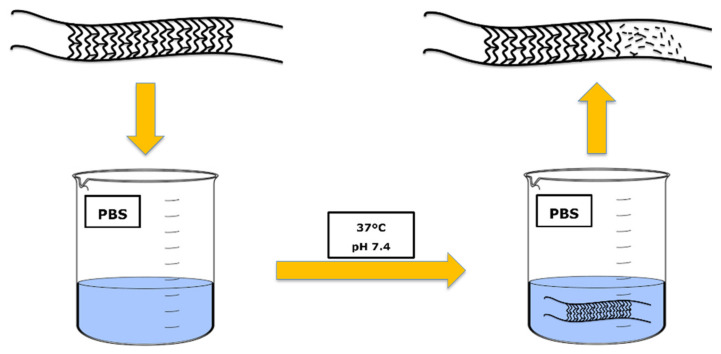
Schematic representation of the in vitro degradation of vascular stents.

When bioresorbable materials are used to manufacture vascular stents, it is necessary to know their degradation profile. It is essential to evaluate the degradation kinetic since it determines the time range during which the mechanical properties are still adequate [30]. The degradation process must consider the type of material, molecular weight, crystallinity degree, and the pH of the surrounding environment [31]. Moreover, the degradation rate is also influenced by the processing technique used to fabricate the vascular stent [32]. Regarding the degradation of bioresorbable stents, several authors have described some in vitro procedures to assess the degradation profile, namely immersion in simulated body fluid (SBF) and immersion in phosphate-buffered solution (PBS) (Figure 2) [33,34].

### 2.2. Types of Vascular Stents

In this review, the description of the vascular stents will be carried out under the most common terms presented by the authors and selling companies to facilitate their understanding. According to the literature, vascular stents can be classified into three distinct groups that have differences in the base material and surface of the stent: bare-metal stents (BMSs), drug-eluting stents (DESs), and bioresorbable stents (BRSs). Despite the differences, all types of stents must share some of the characteristics and properties mentioned in the previous section.

Bare-metal stents were the first devices implanted to treat atherosclerosis; when compared to balloon angioplasty, they reduced the rate of restenosis. Usually, these devices have a permanent metallic structure without drugs loaded on their surface [35]. Due to the mechanical properties of metals, BMS stents have an increased radial strength that allows robust mechanical support to the vessel wall with a thin strut. Devices with reduced struts are known for having a reduced crossing profile, which induces less tissue injury and less disruption in the blood flow [36]. Nevertheless, introducing a permanent metallic framework triggers inflammatory responses, such as neointimal hyperplasia, that lead to the artery’s reblockage and the activation of the coagulation cascade [13]. These events can be associated with the metallic ions released from the stainless steel (316LSS) and cobalt–chromium (CoCr) alloys through the presence of crevice, pitting, and stress corrosion cracking. For instance, the release of nickel, molybdenum, and chromium ions can trigger genotoxic and mutagenic events and activate allergic responses [36]. As a result, drug-eluting stents emerged to overcome the undesirable effects of BMS implantation [8].

Drug-eluting stents are characterized as having a permanent metallic structure with a coating that acts as a drug reservoir. An ideal DES includes the elution of antiproliferative and anti-inflammatory drugs that are released over time, delaying the biological response. The selected drugs must be capable of helping appropriate healing and endothelialization and being efficient in inhibiting platelet aggregation, inflammation, vascular smooth muscle cell (VSMC) proliferation, and migration [37]. Due to the presence of pharmaceuticals, DESs has shown lower ISR rates than BMSs [38].

DESs can be constituted by different materials in the core structure and the surface coating. Usually, the base structure of this type of stent is fabricated with metallic alloys. The coatings can be nondegradable (first generation of DESs) or degradable (second generation of DESs), and each one of them has different mechanisms and times to release the eluting drugs [39]. The first generation of DESs consisted of a permanent metallic framework, a nondegradable polymeric coating, and a pharmaceutical loaded on the coating layer. The degradable polymeric coating is characteristic of the second generation and diminishes the adverse clinical occurrences compared to the first generation of DESs [37]. Concerning the materials used as coatings, the most used are bioresorbable polymers, but a few studies also describe inorganic coatings to be applied in DESs [39]. The arrival of new generations of DESs reduced the biological events, but late IST and ISR events are still active and compromise the long-term efficiency and safety of DESs [40]. Consequently, the concept of BRSs arose.

**Figure 3:**
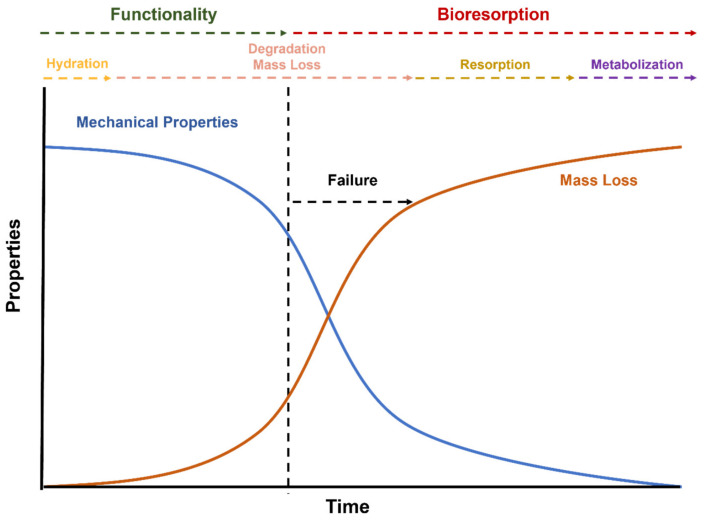
Relationship between polymer properties and degradation of postimplanted BRSs.

Biodegradable stents have been investigated and credited as the fourth revolution in the stenting field as a possible solution for the treatment of coronary artery disease [41]. This type of stent provides temporary scaffolding to the arterial wall and then is degraded and resorbed by the human organism (Figure 3). Bioresorbable stents have either degradable polymers or metals as the base material of the core structure [41]. Compared to metals, polymers have the advantage of being less dense, more flexible, and easy and cost-effective to modify [42].

Usually, the degradation begins in the outer face of the stent, having diffusion of the water into the stent structure and, therefore, chain scissions of the polymeric network. Depending on the polymeric material, design of the invasive implant, and/or environment, the degradation can take from a few months to years [6].

**Figure 4:**
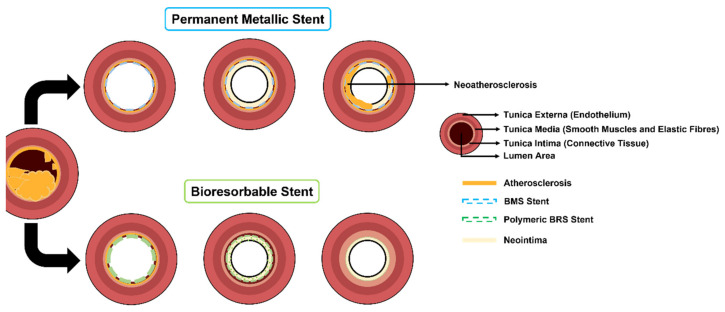
Comparison between bioresorbable stents and permanent metallic-based stents.

Bioresorbable devices offer superior conformability compared with permanent metallic stents while allowing luminal gain and reducing the risk of late ISR and IST associated with DESs. According to Kereiakes et al. [43], several commercial BRSs showed preservation or gain of the lumen in clinical models after the implantation. This phenomenon happens because the bioresorbable struts are replaced by VSMCs and collagen fibers, regulating the lumen area and preventing neoatherosclerosis (defined as the “transformation of stent neointima from normal neointima to an atherosclerotic lesion”). This event is different in permanent metallic stents because, after a time, the lumen area decreases due to the plaque growth or neoatherosclerosis on the surface of the metallic device. The inadequate VSMC endothelization causes neoatherosclerosis on DESs or BMSs, triggering ISR and IST after a while [43,44] (Figure 4).

Despite all the advantages, BRSs present some drawbacks compared to base materials. In the case of polymers, besides the lack of radiopacity, they present low stiffness and low radial strength. Thus, they need thicker struts to offer equivalent support to the vessel compared to BMSs or DESs [37]. Additionally, the lack of radial strength can lead to radial elastic recoil [38]. Toong et al. [24] demonstrated in their study that thicker struts can originate shear/laminar blood flow disruptions, reducing endothelialization and increasing thrombogenicity. In order to thwart these events, metallic-based BRSs have emerged, providing better mechanical strength than polymers. However, they have a faster degradation than polymeric devices in environments with chloride (blood), leading to a higher probability of adverse biological events after implantation [41]. Thus, future research must study ways to improve the mechanical features of polymeric bioresorbable stents due to their higher cellular compatibility and advantageous degradation time compared with metallic materials [45].

The present manuscript will focus on polymeric bioresorbable stents, providing a detailed description of the main polymers used for stent fabrication.

## 3. Polymeric Materials for Bioresorbable Vascular Stents

Bioresorbable stents are made with materials that can be degraded and absorbed by the human organism, entering the main metabolic pathways, such as the Krebs cycle. The main objective of bioresorbable materials is to provide temporary support to the vessel during the healing time and then be eliminated by the body, leaving the artery with a healthy endothelium and normal blood flow. As mentioned previously, the absence of foreign material will decrease the risk of late ISR and IST [41]. In addition, significant groups of patients, such as children, the elderly, or diabetics, who suffer from problems involving several repeated surgeries will benefit from this technology since removing the implant is unnecessary [39].

Currently, polyesters are used to manufacture BRSs because of their tailorable biodegradability. Polymers such as poly(lactic acid) (PLA) and its enantiomers, poly(ε-caprolactone) (PCL), poly(glycolic acid) (PGA), and poly(lactic-co-glycolic acid) (PLGA), are used in polymeric-based cardiovascular stents [41]. This section will address some aspects of these polymers, including their degradation and/or reabsorption processes.

### 3.1. Poly(lactic acid)

PLA is accepted by several regulatory agencies as a safe, biodegradable material to be applied in numerous medical applications [46]. Amongst all polymers suggested and applied for fabrication of vascular stents, by far the most common is PLA due to its optimal combination of cellular compatibility, degrading pattern, and mechanical strength [23,47].

**Figure 5:**
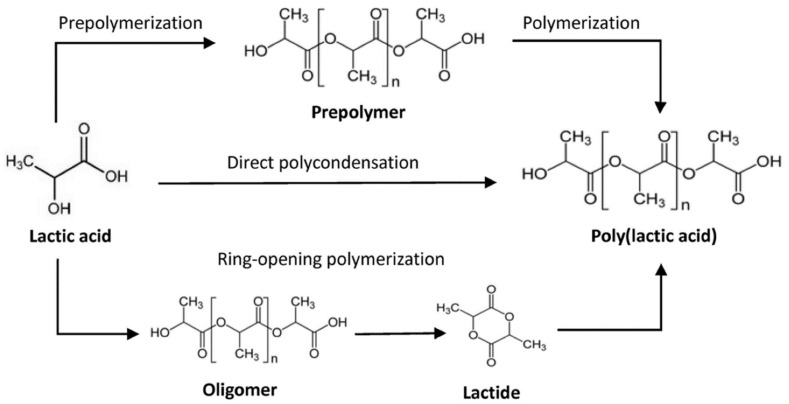
Routes of poly(lactic acid) (PLA) synthesis from lactic acid [53].

PLA is acquired and synthesized from lactic acid or lactide. This compound is considered a renewable source since it is extracted from corn starch or sugar cane [48,49]. The biodegradable polymer can be synthesized using different routes: ring-opening polymerization (ROP) or polycondensation of lactide (Figure 5) [50,51]. ROP is performed using heterocyclic monomers with at least one ester bond in the ring, and polycondensation is conducted using diols or hydroxy acids and diacids [52]. PLA can have two enantiomers (L-lactic acid and D-lactic acid) generally produced by fermentation. Additionally, it is possible to synthesize a racemic mixture denominated LD-lactic acid in an equimolar mixture of L and D enantiomers, showing different properties and characteristics from the individual ones [50]. To sum up, the PLA can be synthesized as PLLA, PLDA, and PDLLA. PLA designation is used when it is not known if the polymer is a pure enantiomer, a mixture, or their proportion.

The literature shows that the different enantiomers of PLA produce different characteristics and properties in vascular stents. The percentage of these compounds determines properties such as glass transition and melting temperatures or percentage of crystallinity which will affect the mechanical properties and the biodegradation rate of the stent [6]. For instance, PDLLA has lower tensile strength and higher degradation time than PLLA and PDLA [54]. In what concerns the application in vascular stents, the PLA can also be copolymerized with other polymers such as PGA or PCL to improve the performance of the stents. Compared to other polymeric materials, PLLA has better mechanical properties than PCL and a lower degradation time than PLGA (Table 2).

**Figure 6:**
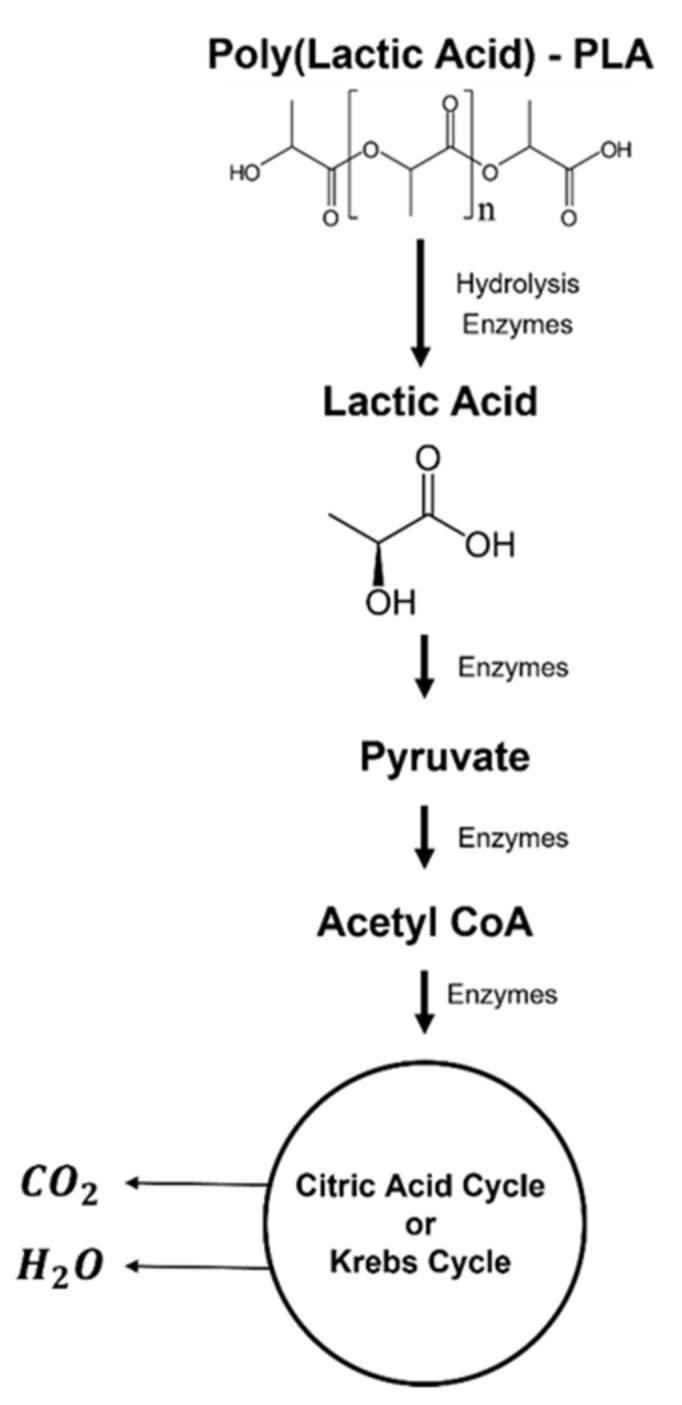
PLA biodegradation path.

The degradation of this material starts with the diffusion of water molecules into the polymeric chain. After, there is a decrease in the molecular weight due to the hydrolysis that breaks the ester bonds. Over time, this process leads to the fragmentation of the polymer, causing mass loss and reducing the mechanical strength. The breaking of the ester bonds leads to the formation of lactic acid [46,55]. Lastly, this product is converted into pyruvate, which enters the Krebs cycle, and CO_2_ and H_2_O are originated as final products, being eliminated from the body via lungs or kidneys (Figure 6) [56].

PLA has a hydrophobic nature due to the presence of the methyl group, making the material more resistant to hydrolysis when implanted [57]. The hydrolysis and degradation profiles depend highly on the stereochemistry, molecular weight, crystallinity percentage, and local pH [46,58]. Moreover, the design of the vascular stents can affect the degradation rate since the diffusion and distribution of water or body fluids into the polymeric network will vary according to the morphology and porosity inside the device. During the degradation of the medical device, the mechanical properties will be lower, and thus, it is necessary to guarantee enough time for the artery to heal appropriately. Some studies have suggested reinforcement with fibers, polymerization with other polymers, or new manufacturing techniques that allow tailoring the properties [6,59].

### 3.2. Poly(glycolic acid)

**Figure 7:**
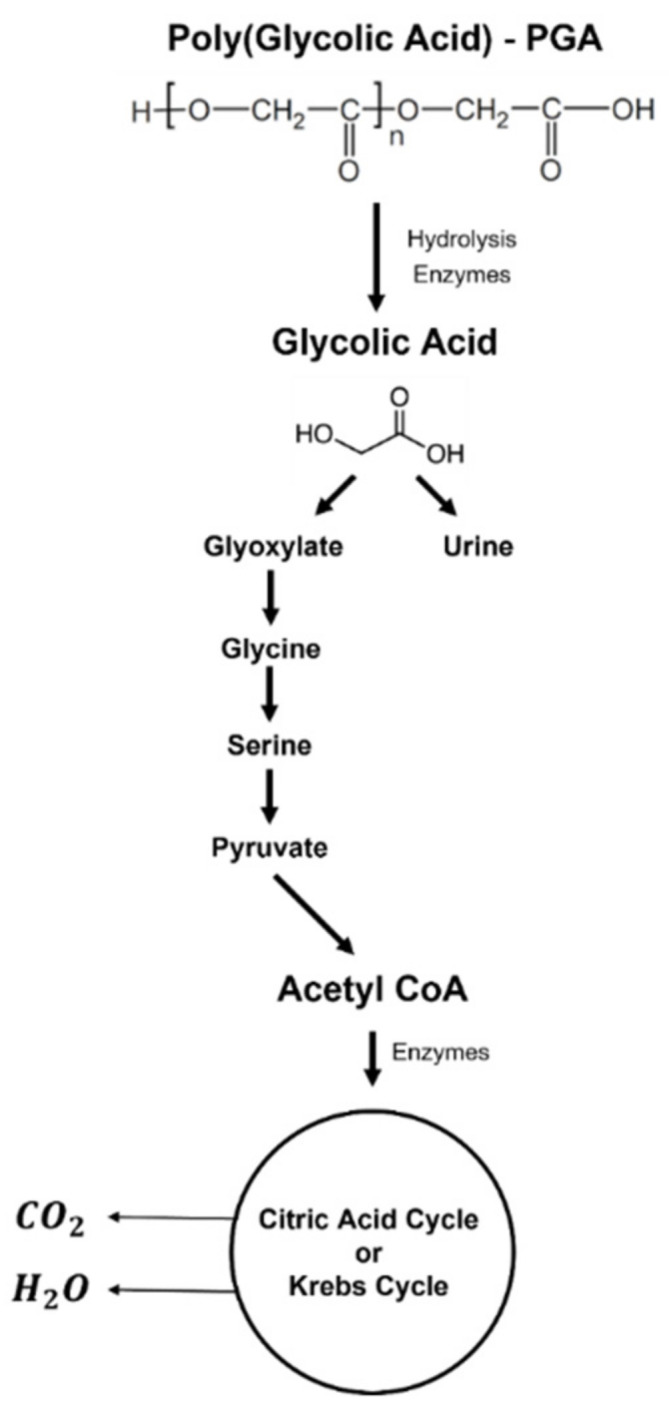
Biodegradation process of PGA.

Poly(glycolic acid) is a biodegradable polyester accepted for medical application as an absorbable suture material [49,60]. Due to the fast degradation of PGA, it is a challenge to synthesize this polymer. The synthesis of PGA is conducted using the monomers glycolic acid and glycolide (cyclic dimmer of glycolic acid) (Figure 7). This biodegradable polymer can be produced using ROP of glycolide or polycondensation of glycolic acid. Despite its characteristics, the synthesis of PGA remains extremely expensive compared to that of PLA [61].

For PGA, as for almost all polymers, the mechanical properties vary with crystallinity degree [49]. Compared to other polymers used for vascular stent manufacturing, such as PLLA and PLGA, PGA presents better tensile strength and stiffness, as demonstrated in Table 2. Nevertheless, this material presents low ductility, and the difficulty in handling this polymer makes PGA inappropriate for BRS manufacturing [41]. Moreover, the synthesis of this polymer is expensive compared to other materials, which is another disadvantage when applied in polymeric bioresorbable stents.

Like other polyesters, the degradation of PGA starts with the hydrolysis of ester linkages. In the first stage, the water molecules diffuse into the polymeric network. Then, the hydrolytic chain scission occurs in the amorphous domains, while the crystalline regions suffer hydrolytic degradation [62]. The resulting monomer is glycolic acid, which is metabolized through several metabolic pathways. The final metabolites are excreted via urine and respiration (Figure 7).

The degradation rate is highly dependent on several factors, including local pH, crystallinity percentage, average molecular weight, and hydrophilicity. According to the literature, PGA is considered more hydrophilic than PLA. The absence of the methyl asymmetrical groups facilitates the hydrolysis of the PGA, which loses 60% of its mass throughout the initial two weeks [63]. Although several authors suggest using PGA as a base material for vascular stents due to its mechanical properties, PGA is unfavorable. The fast degradation of PGA does not allow adequate vessel healing and may lead to a decrease in pH, which, in turn, triggers biological responses [60,64].

### 3.3. Poly(lactic-co-glycolic acid)

**Figure 8:**
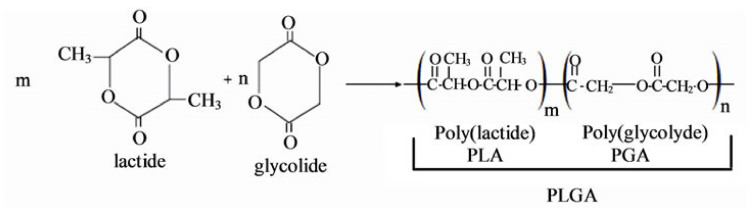
Chemical structure of the cyclic dimers and the copolymerization reaction (ROP) [65].

Poly(lactic-co-glycolic acid) or PLGA is a copolyester synthesized through the copolymerization of PLA and PGA [54]. This copolymer offers the ability to tailor several properties and characteristics, e.g., mechanical properties, wettability, and degradation rate, by changing the PLA/PGA ratio [60]. Usually, PLGA is copolymerized by two main processes: (1) direct polycondensation of glycolic and lactic acids and (2) ROP of cyclic lactide and glycolide (Figure 8) [62].

The degradation process of PLGA also involves the hydrolysis of ester bonds, originating lactic acid and glycolic acid that are eliminated through the metabolic pathways. PLGA has a slower degradation time than PGA due to the methyl group of the lactic acid that is more hydrophobic (Table 2). This characteristic can be tailored by adjusting the LA:GA ratio, the lactide stereoisomeric composition (D, L, or DL), the monomer sequence, and the end group of the copolymer [54,62].

### 3.4. Poly(ε-aprolactone)

**Figure 9:**
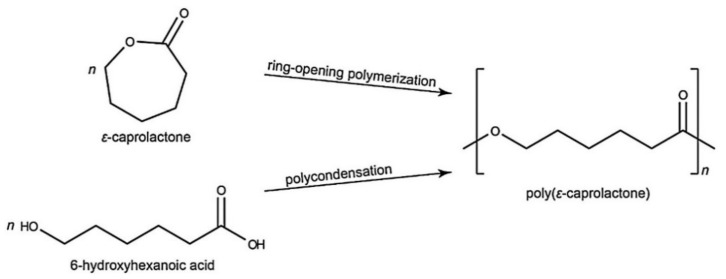
Scheme of the synthesis of PCL from ε-CL and hydroxyhexanoic acid. Reprinted with permission from Elsevier [67].

Another polymer that is very well described in the literature to apply in bioresorbable stents is poly(ε-caprolactone). PCL is a hydrophobic and semicrystalline polyester approved by the FDA for several medical applications. PCL can be synthesized through ROP of the cyclic monomers of ε-caprolactone (ε-CL) in the presence of catalysts or polycondensation of a hydroxycarboxylic acid [66] (Figure 9).

The properties of PCL vary according to the crystallinity percentage and molecular weight, the percentage of crystallinity being higher when the molecular weight is lower [46]. In addition, the properties can differ when tailored for a specific application or using different manufacturing techniques, for example, by changing the printing parameters of 3D-printed vascular stents [32,67]. Compared to PLA, PCL has greater flexibility but meaningfully inferior strength and slower absorption time (Table 2) [41,68]. Therefore, it is not an appropriate base material to apply in vascular stents considering the mechanical properties. Recently, PCL has been blended with PLA to improve the properties of vascular stents (Table 2) [33].

**Figure 10:**
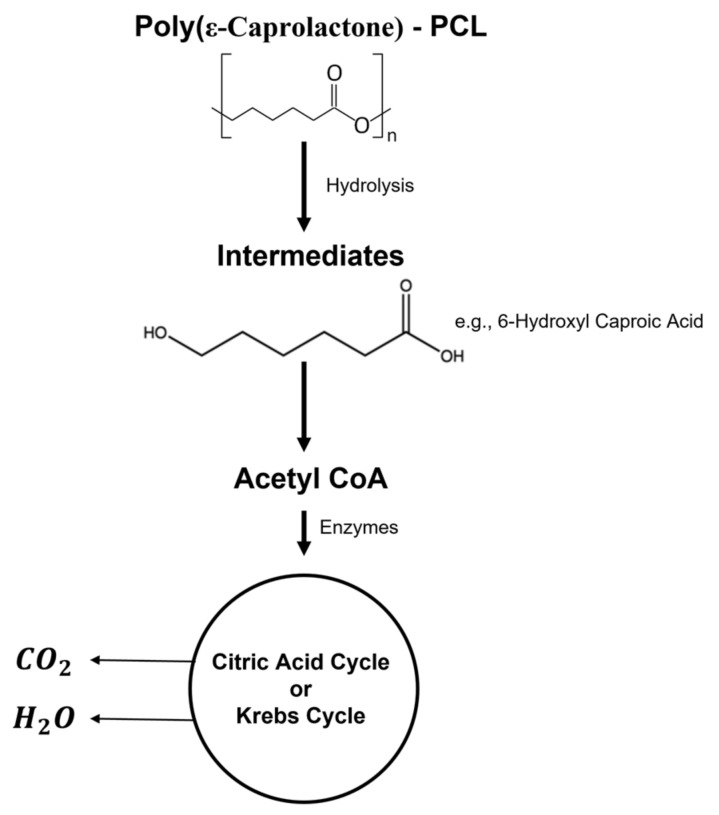
Biodegradation process of PCL.

The nonenzymatic degradation or hydrolysis of the ester groups is the most common way to degrade PCL. The degradation begins with the diffusion of water molecules that triggers the random hydrolytic cleavage of the ester bonds in the polymeric network, causing a decrease in the molecular weight. Then, the macrophages capture the small PCL fragments to be degraded and eliminated by the body. The main intermediates or metabolites that result from PCL degradation are acetyl CoA and 6-hydroxyl caproic acid, which are eliminated from the body through the citric acid cycle (Figure 10) [69].

**Table 2:** Properties of some polymers used for biodegradable stents (adapted from [54,64]).

Polymer	T_g_ (°C)	T_m_ (°C)	Young’s Modulus (GPa)	Tensile Strength (MPa)	Elongation at Break (%)	Degradation (Months)
PDLLA	55		1–3.5	40	1–2	3–4
PLLA	60–65	175	2–4	60–70	2–6	>24
PGA	35–40	225–230	6–7	90–110	1–2	4–6
PDLGA (50/50)	45		1–4.3	45	1–4	1–2
PLGA (82/12)	50	135–145	3.3–3.5	65	2–6	12–18
PCL	−54	55–60	0.34–0.36	23	>4000	24–36
PLA/PCL (70/30)	20	100–125	0.02–0.04	2–4.5	>100	12–24
PC	~147	225	2–2.4	55–75	80–150	>14

### 3.5. Other Polymers

Several other polymeric materials have been suggested as base materials for bioresorbable vascular stents. Polymers such as poly(desaminotyrosyl-tyrosine ethyl ester) carbonate (PTD-PC) and salicylate/poly(lactide anhydride) (SA/AA) are examples that have been developed by some pharmaceutical companies [36].

PTD-PC-based stents are bioresorbable devices commercialized by REVA Medical Inc. PTD-PC is a polymeric material that contains a tyrosine polycarbonate backbone linked to iodine to give radiopacity to the device. According to the literature, this device presents better mechanical properties than other polymeric-based devices such as PLA-based stents. According to McMahon et al. [47], PTD-PC and PLA devices present tensile strengths of 60–220 MPa and 50–60 MPa, respectively. The PTD-PC scaffold is characterized by not having a coating on the surface, and its biosorption time varies between 6 and 48 months [47,70]. The degradation products of this type of material are carbon dioxide, water, ethanol, and iodinated desaminotyrosyl-tyrosine, which the body will further excrete through metabolic pathways [70].

Another company that uses different polymeric materials to fabricate a vascular stent is Xenogenics Corp. This pharmaceutical corporation used a mix of a poly(lactide anhydride) and salicylic acid (SA) and used sebacic acid as a base material [47]. This material is biocompatible and hydrophobic but degrades faster than other polyesters, and the degradation rate is controlled by the number of anhydride linkages [6,47]. The resulting degradation products have the advantage of reducing the percentage of restenosis.

Regardless of the polymeric material used for the fabrication of vascular stents, it is well known that it must include higher mechanical properties, cellular compatibility, and an appropriate degradation time. Hence, polymeric-based stents need thinner struts and reduced luminal area to avoid flow disruption and other biological events. Apart from the material choice, one possible strategy to overcome the limitations of polymeric BRSs is to change the design of the device or the manufacturing features.

## 4. Manufacturing Technologies

Although the materials’ properties and design features are essential for the successful performance and degradation of BRSs, the manufacturing processes must also be considered. Over the past few years, several technologies have been applied for metallic and polymeric materials, including top-down and bottom-up approaches.

Currently, laser cutting is the most used technique to fabricate the core structure of stents. This approach has been the first choice for the last three decades, fulfilling the design criteria [71]. The main advantage of this manufacturing methodology is related to the production of fragile hollow tubes with small thicknesses and great outline accuracy [72]. Despite its exceptional accuracy and precision, laser cutting is a thermal process that can lead to structural problems such as residual tensions, microcracks, or, more commonly, heat-affected zones. Another point to be highlighted is that this technology is a subtractive method, which implies that sustainability is not high due to the amount of waste production. Additionally, the posttreatment of the surface will increase the price of the stents even more [73].

Several innovative processes have been developed as an alternative to overcome the problems associated with laser cutting. Numerous scientific studies suggested using AM as a solution. Several studies mentioned that 3D-printed structures for clinical practice remain challenging in cardiovascular medicine. Further research is needed to exploit the true potential of additive manufacturing in surgery [74]. It is estimated that AM could be transformational, and some of the goals include creating multidisciplinary teams with physicians and engineers implementing rigid guidelines and appropriate criteria [75].

Regarding the fabrication of cardiovascular stents, AM allows the fabrication of customized vascular stents and decreases the manufacturing costs compared to conventional techniques [76]. By definition, all AM technologies can be combined with several imaging techniques to produce more realistic and accurate 3D models. Some examples of coronary imaging are coronary angioplasty, magnetic resonance, and, more recently, intracoronary optical coherence tomography that considers the blood vessels’ morphology and characteristics, allowing a better design of the vascular stent [27]. Demir and colleagues [77] used selective laser melting (SLM) to produce CoCr stents. Although it proved to be an alternative solution, SLM has a higher processing temperature, creating poor surface finishing and material damages similar to laser cutting [32].

More recently, fused filament fabrication (FFF) has been proposed to manufacture vascular stents, namely polymeric BRSs. Compared to other AM technologies, FFF has some advantages, including lower processing temperature and a growing “library” of polymers and polymer-based (nano)composites that can be used, in comparison with SLM and stereolithography (SLA) [78].

Four-dimensional printing is a newly emerging topic associated with AM. In 4D printing, the printed samples can change their shape when an external stimulus is applied [79]. Many shape memory polymers described in the literature can be programmed to respond to a stimulus after printing. Some examples of materials are PLA, PCL, and poly(ethylene glycol) (PEG), which are examples of materials that can be applied to the manufacture of vascular stents [76,80]. Prior research suggests that 3D printing stents using shape memory polymers could offer a patient-personalized production, removing the requirement for balloon expansion, reducing the possibility of stent displacement, and leading to a new age for stent technology [76,81,82].

Jia et al. [83] proposed using FFF to create a polymeric BRS using a shape memory polymer, PLA. After the production of the vascular stent, the device can keep the compacted shape at ambient temperature and then self-expand through temperature stimulus [83]. Another study mentions the use of FFF as a technique to fabricate BRSs based on PCL, also a shape memory polymer. This approach can create a safer, custom-made, and more appropriate device [32,33]. Nevertheless, it is necessary to improve the mechanical strength of the devices made with these polymers.

## 5. Processing Parameters in AM

It is known from previous studies that the characteristics and properties of the bioresorbable thermoplastic polymers can suffer changes during the manufacturing process. FFF is a technique with numerous parameters that influence the quality of the 3D-printed parts, such as the mechanical properties and the degradation rate [84]. Many questions remain unanswered about the effect of the printing parameters on vascular stents. To address these questions, we address the influence of the main printing parameters, such as layer thickness, raster angle, raster width, build orientation, infill pattern, and infill density.

**Figure 11:**
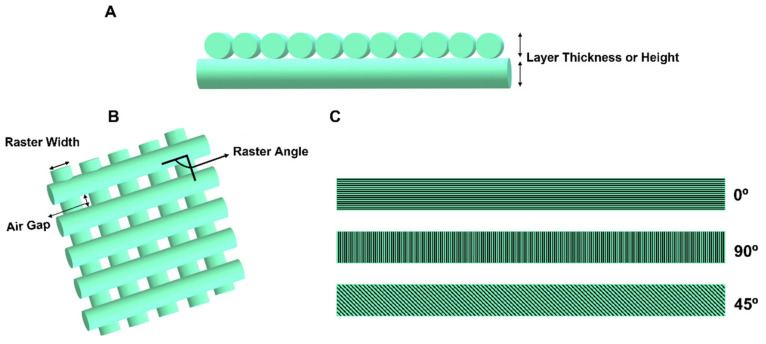
Schematic representation of some FFF printing parameters: (**A**) layer thickness; (**B**) air gap, raster orientation, and raster width; (**C**) examples of raster angles.

The layer thickness or layer height is one of the most reported printing parameters in the literature that affect the properties of the printed parts. In the FFF technique, the sample is printed layer by layer, where each layer has thickness or height measured on the Z-axis (Figure 11A). The thickness is directly correlated with the time of fabrication of the device. A lower thickness increases the number of layers needed to print the specimen, consequently increasing the time of printing [85]. The lower thickness also induces a smoother surface finish, influencing the cellular response after implantation.

Another parameter that is often reported in the literature is the raster angle. This parameter is defined as the angle between the nozzle path and the X-axis of the printing surface (Figure 11B). The raster angle defines the mechanical properties of the printed samples and the accuracy and quality of the printed sample [85]. The value of the raster angle can vary between 0 and 90°, as demonstrated in Figure 11C. It is well known that 3D-printed devices often present anisotropic mechanical properties. Therefore, it is of extreme importance to consider the raster angle to optimize the properties according to the mechanical solicitation of the stent.

The air gap is defined as the gap in the middle of two adjacent rasters on the same layer and is influenced by the raster angle and thickness of the layer (Figure 11B) [86].

**Figure 12:**
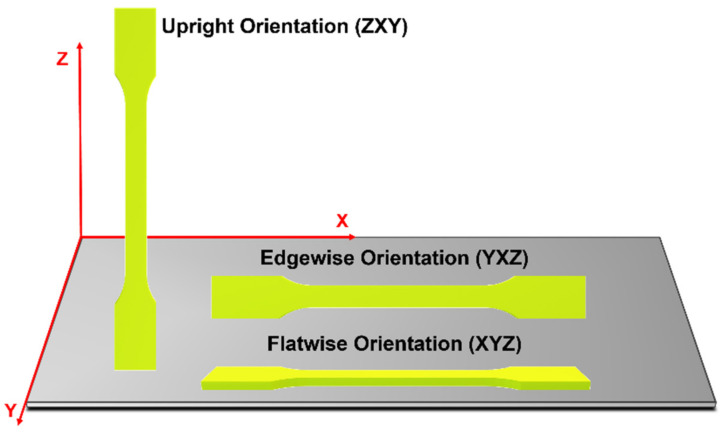
Build orientation.

Several authors also mention the build orientation during the FFF process as an important parameter for tailoring mechanical properties [87]. Build orientation refers to the position (direction and inclination) of the specimen in the build platform. Considering the X-, Y-, and Z-axes, the three main build orientations are flatwise, edgewise, and upright [86,88] (Figure 12). Some authors highlighted the importance of build orientation on features such as build time, manufacturing cost, surface quality, geometric accuracy, and structural and mechanical properties. Besides, Chacón et al. [88] mentioned that the anisotropy of the objects is highly dependent on build orientation which will affect the final properties of the printed sample.

**Figure 13:**
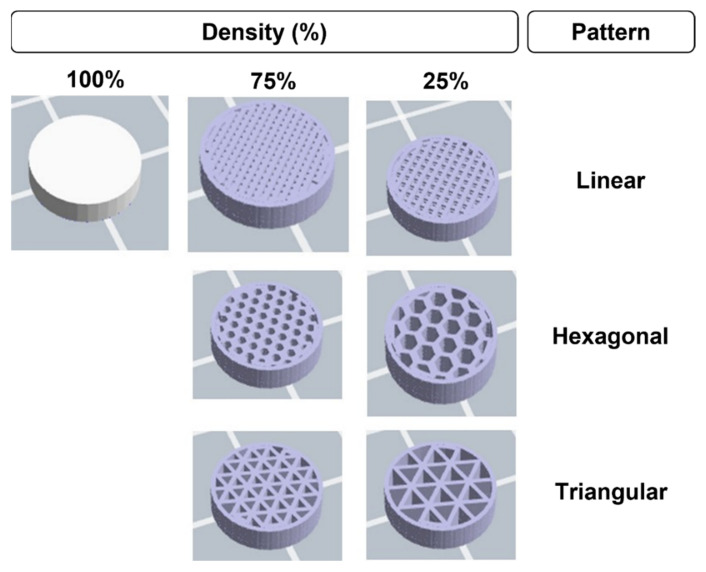
Examples of infill patterns and percentages.

The infill density and pattern are printing parameters with great importance that can affect both mechanical properties and degradation rate. The infill density is described as the amount of printed material within a structure. This parameter is measured in a percentage that varies between 0% and 100%, 0% indicating a hollow part and 100% indicating a filled part. According to the literature, the infill density directly influences the weight, printing time, and cost of the 3D-printed specimen. The infill pattern is the geometry or shape of the printed material inside the structure. The patterns range from the simplest (linear or rectilinear) to the most complex geometries, such as hexagonal or honeycomb geometries. The design of the pattern inside the specimen affects the mechanical properties. This feature can be advantageous when printing a vascular stent [85,89,90]. Figure 13 shows different examples of infill patterns and densities.

### 5.1. Influence of Printing Parameters

The printing parameters affect the characteristics and properties of the degradable polymeric devices [91]. For example, the porosity existing inside the printed device can be controlled [92]. Recently, Chen et al. [30] showed that the porosity and internal architecture affect the degradation profile by interacting and altering the diffusion speed and path of the water molecules during the degradation process. The porous structure offers a higher contact area that allows faster hydrolysis and, consequently, degradation and resorption of the material [30]. On the other hand, the inner structure of printed specimens also affects the mechanical properties [93]. In their study, Singh et al. [55] found that specimens with high compressive strength have a higher degradation time due to the lower porosity inside the printed specimen.

This topic may constitute the object of future studies involving the influence of the printing parameters during the fabrication of polymeric BRSs. As far as we know, only Guerra et al. [32] have studied the influence of the printing parameters in the fabrication of vascular stents. However, they only studied the influence of nozzle temperature, fluid flow rate, printing speed, and printing trajectory. Thus, it is necessary to study many printing parameters to understand their influence on mechanical strength, radial strength, and the time of the material bioresorption. The effects of layer thickness, build orientation, raster angle, and infill parameters will require detailed investigation in vascular stents. This section states the effect of the mentioned printing parameters on the mechanical properties and degradation profile, correlating with the vascular stents.

#### 5.1.1. Layer Thickness

Previous studies have emphasized that layer thickness has an essential role in the mechanical properties of 3D-printed samples. According to several authors, a lower layer thickness is recommended to achieve better mechanical performance. It is suggested that a small layer thickness enhances the tensile strength and the elastic modulus [94]. A study from Kovan and coworkers [95] indicated that as the thickness of the layer decreases, the resistance to load increases. This suggests that the vascular stents should be printed with minimum layer thickness to achieve better radial strength and support the load exerted by the artery wall. The bending strength is also affected by the layer height, and Sousa et al. [57] showed that the lower the layer thickness, the higher the bending strength. These results highlight an essential aspect because the vascular stent must be able to bend to be well fitted to the blood vessel [23].

It was reported in the literature that the mechanical properties of FFF parts could be compromised due to the presence of pores. A pore is created during the bonding of the layers. According to Garzon-Hernandez et al. [96], the internal porosity of a specimen can increase 3.8 times if the layer height increases from 0.1 to 0.3 mm. These results are a consequence of the decrease in the layer thickness that favors cohesion between layers, diminishing the porosity [57]. The decrease in the porosity of the stent structure will lead not only to a lower degradation rate but also to better mechanical properties. In the case of polymeric BRSs, no studies have addressed the influence of layer height on the mechanical properties and degradation profile of 3D-printed devices.

#### 5.1.2. Build Orientation and Raster Angle

Several studies have been proved that samples produced through FFF present an anisotropic behavior caused due the layer-by-layer process. The anisotropy is highly affected by the orientation of the layers and, consequently, influenced by the build orientation during the printing procedure [97]. The most used orientations in the literature are longitudinal directions (flatwise and edgewise) and transversal direction (upright). These orientations produce significant differences in the morphology and structure of the 3D-printed specimens and, therefore, different mechanical properties [95,98]. Thus, it is necessary to consider this parameter during the fabrication of vascular stents.

Regarding the mechanical properties, it is demonstrated in several studies that the vertical direction presents the poorest mechanical properties compared to longitudinal building orientations [87,99]. Chacón and colleagues [88] stated in their manuscript that the vertical direction showed that lowest value of tensile strength and stiffness compared to the other directions. Likewise, Ashtankar et al. [100] observed that when the build orientation is flatwise, the tensile and compressive stresses are 23.68% and 16.65% greater than the values for the vertical direction, respectively. Furthermore, it is described that the longitudinal samples present a translayer failure and a ductile fracture while the transversal sample presents an interlayer failure and a more brittle fracture [88]. Another characteristic to take into account during stent manufacturing is flexibility. According to the literature, the flexural properties of samples printed in upright build orientation are worse than those of specimens printed with longitudinal orientations. The authors stated that vertical printed specimens suffered failure at lower deflection values [97].

**Figure 14:**
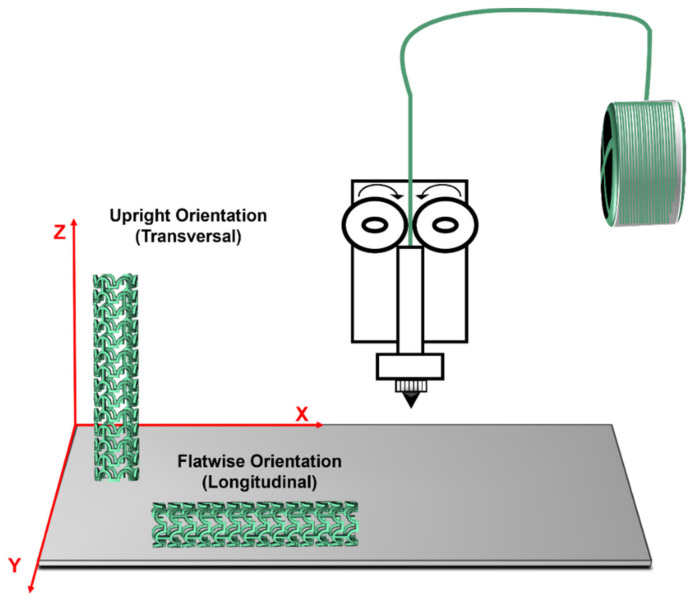
Schematic representation of the building orientation of 3D-printed vascular stents.

It has been shown that the building direction or orientation is an essential parameter to consider during the fabrication of vascular stents through FFF. The polymeric bioresorbable stent must have the best mechanical performance to support the load exerted by the artery wall. Moreover, the implant must resist the compression from the vessels during the heartbeat movements [23,24]. According to the information stated previously, the ideal mechanical performance of the stent will be achieved with the longitudinal printing direction (flatwise or edgewise) since it will possibly combine the best radial strength with the best flexural properties (Figure 14). Nonetheless, the samples printed in longitudinal directions have more irregularities on their surfaces than transversally printed samples. The area of the underside layer in contact with the 3D-printer platform is higher for the samples printed in the longitudinal direction, creating more irregularities on the surface of the printed samples [98]. Although this can influence the degradation rate of the material, it also will allow the appropriate cell attachment and proliferation, granting adequate healing of the damaged artery [55,101].

The build orientation influences the raster angle, impacting the morphology and mechanical performance of a sample printed by FFF [85]. The variation of the tensile properties with this parameter occurs because the raster angle influences the direction and transference of the load inside the printed sample. It is described that an increase in the raster angle leads to a decrease in the tensile properties [99]. According to several authors, to improve the mechanical properties and reduce the anisotropy of the printed part, it is suggested to print with a raster angle of 45°/−45° [92,99]. Haryńska and coworkers [102] demonstrated that the tensile strength is higher for an angle of 45°/−45° compared to the raster angles of 0 and 90°. In addition, the authors explained that this happens because of the greater tension of the layers positioned perpendicularly to the stretching direction of the printed specimen [102].

The samples with a raster angle of 90° present the best results regarding the compressive strength. Nevertheless, the 45°/−45° specimens revealed an increased capacity to bear cyclic tensile loading [99]. Although compressive strength is an important factor in the fabrication of stents, the dynamic mechanical behavior presents higher importance when the device is implanted. As mentioned previously in the requirements for vascular stents, the bloodstream induces cyclic stresses (cyclic loads), which lead to fatigue failure and compromise the long-term durability of the device [16,27]. Thus, the best raster angle to be applied when printing vascular stents is 45°/−45° in order to ensure the stability and durability of vascular stents after implantation.

#### 5.1.3. Infill Parameters

The infill parameters have great importance in tailoring the mechanical properties and the degradation profile of printed devices. The infill density or percentage is the parameter with the most important role in controlling the factors mentioned before. Some studies showed that mechanical properties are more influenced by the infill percentage than by the build orientation or layer thickness [103]. For all infill patterns, it is consensual that the tensile properties increase as the infill density increase [104]. Research from Culbreath et al. [84] stated that a rise in the infill percentage leads to a higher tensile strength value. In addition, the flexural proprieties are dependent on the infill percentage, increasing as the infill density increases [105]. This phenomenon happens due to the higher quantity of material inside the structure to support a higher load [85].

In the case of polymeric BRSs, a high tensile strength helps accomplish a sufficient radial strength to resist the forces exerted by the vessel wall. In addition, the stent must have enough flexural strength to support the bending forces [24,25]. Thus, when using the FFF technology, it is recommended to print a vascular stent with the maximum infill percentage to achieve the best combination of mechanical properties.

When considering bioresorbable stents, it is necessary to evaluate the degradation of the material. In the case of a lower infill percentage, the number of pores inside the printed structure will be higher (Figure 13). Consequently, it will facilitate the degradation process due to the greater contact area that easily allows the diffusion of water molecules and, therefore, the hydrolysis of the material and its resorption.

Nam et al. [106] tested the recovery of a device printed with PLA with different infill percentages. It was proved that a higher infill density allowed a better shape recovery [106]. Once again, the maximum infill percentage seems to be the appropriate choice to produce polymeric BRSs that respond to an external stimulus to self-expand inside the artery. Further investigation using polymeric BRSs is needed, representing an essential opportunity for advancement in the research field of 4D printing in the medical industry.

The infill pattern determines the shape inside the structure, controlling the raster and bonding between layers [85]. A few studies have explored the influence of infill patterns on mechanical properties. For instance, Dezaki et al. [105] mentioned in their study that devices printed with a honeycomb pattern presented the best mechanical properties, namely tensile strength. On the other hand, Akhoundi and Behravesh [13] concluded in their research that a triangular infill pattern had better tensile strength when compared with a honeycomb. In addition, Algarni et al. [103] compared different infill patterns and concluded that this parameter significantly influenced fatigue life. There is still a lack of consensus regarding the adequate pattern that provides the best mechanical performance. Further work is essential to disentangle these complexities related to the infill pattern, and questions remain regarding the effect of the infill pattern on the stability and mechanical performance of 3D-printed polymeric BRSs.

In conclusion, there are some potentially open questions about optimizing several printing parameters to produce polymeric bioresorbable stents through FFF with the best mechanical performance during the healing time and artery recovery.

## 6. Conclusions and Future Perspectives

Polymeric BRSs are a promising approach for vascular stenting in specific situations. For these devices, the conjugation of the appropriate mechanical properties with the adequate cellular response is the golden rule for optimal performance. Besides the properties or characteristics of the polymeric material and the design of the devices, the manufacturing technique may also influence the device’s performance. Additive manufacturing techniques arise because they conjugate complex geometry, personalization, and reduced raw material use and waste production. Among AM technologies, FFF is arising as one of the best choices to attain these objectives.

Despite the exciting advances in FFF in healthcare, plenty of challenges remain. The number of 3D-printable materials is growing, but 3D-printable and implantable devices suited for the fabrication of stents lack FDA approval. Besides, the absence of well-defined FDA guidance for printing parameters and testing procedures is problematic for researchers. As far as we know, there is still no 3D-printed cardiovascular stent with the approval of the FDA for the treatment of atherosclerosis. Future research should consider the potential effects of printing parameters in vascular stent fabrication, such as printing patterns’ influence on the mechanical properties and degradation profile. Therefore, the printing parameters for the main base materials used in polymeric BRSs should be studied by studying either single-material or multimaterial samples. Many recommendations for future research are given throughout this manuscript. This area is one of the tough challenges for all academics making 4D-printed vascular stents an open field to explore.

## Figures and Tables

**Table 1:** Requirements and properties that devices (stents) and materials must satisfy to avoid failure (adapted from [16]).

Requirement	Description
High radial strength	Radial strength plays a crucial role in preventing the recoil of the stent by providing radial or structural support to the vessel (ASTM F3067-14).
Low elastic radial recoil	In order to attain a fixed final diameter of the stent appropriate for the host artery diameter, the property of low elastic radial recoil is of importance (ASTM F2079-09).
Good flexibility	For the proper placement of the stent in the tortuous geometry of blood vessels, good flexibility of the designed stent is essential to place it with the help of a catheter (ASTM F2606).
Minimal stent profile	During implantation, to avoid the unnecessary disturbance of blood flow, it is desirable to have a minimal stent profile.
Minimal foreshortening	During the expansion of the vessel, the precise placing of the stent is important; hence, it should possess minimum foreshortening.
Cellular compatibility	The stent material must not cause any adverse reaction or injury in the human body, so cellular compatibility is crucial.
Radiopacity	For delivering the stent at the appropriate position, the radiopacity of the material must be considered.
Excellent fatigue properties	The blood flow induces cyclic stresses, and hence, due to the application of this cyclic load, fatigue failure in the material drastically increases. The selection of the stent material is such that it can withstand a minimum of 380 million cyclic load means up to 10 years (ASTM F2477-07).

## Data Availability

Not applicable.

## References

[1] Roth G.A., Mensah G.A., Johnson C.O., Addolorato G., Ammirati E., Baddour L.M., Barengo N.C., Beaton A.Z., Benjamin E.J., Benziger C.P. (2020). Global Burden of Cardiovascular Diseases and Risk Factors, 1990–2019: Update From the GBD 2019 Study. J. Am. Coll. Cardiol..

[2] Amani S., Faraji G., Kazemi Mehrabadi H., Abrinia K., Ghanbari H. (2017). A combined method for producing high strength and ductility magnesium microtubes for biodegradable vascular stents application. J. Alloys Compd..

[3] Behera S.S., Pramanik K., Nayak M.K. (2015). Recent Advancement in the Treatment of Cardiovascular Diseases: Conventional Therapy to Nanotechnology. Curr. Pharm. Des..

[4] Dahlöf B. (2010). Cardiovascular Disease Risk Factors: Epidemiology and Risk Assessment. Am. J. Cardiol..

[5] Institute of Medicine (US) Committee on Social Security Cardiovascular Disability Criteria (2010). Cardiovascular Disability: Updating the Social Security Listings.

[6] Bink N., Mohan V.B., Fakirov S. (2021). Recent advances in plastic stents: A comprehensive review. Int. J. Polym. Mater. Polym. Biomater..

[7] Ringer A.J., Hopkins L.N., Aminoff M.J., Daroff R. (2003). Endovascular Therapy. Encyclopedia of the Neurological Sciences.

[8] Blair R.W., Dunne N.J., Lennon A.B., Menary G.H. (2019). Multi-objective optimisation of material properties and strut geometry for poly(L-lactic acid) coronary stents using response surface methodology. PLoS ONE.

[9] Wache H.M., Tartakowska D.J., Hentrich A., Wagner M.H. (2003). Development of a polymer stent with shape memory effect as a drug delivery system. J. Mater. Sci. Mater. Med..

[10] Pan C., Han Y., Lu J. (2021). Structural Design of Vascular Stents: A Review. Micromachines.

[11] Qiu T., Zhao L. (2018). Research into biodegradable polymeric stents: A review of experimental and modelling work. Vessel Plus.

[12] Park J., Kim J.K., Park S.A., Lee D.W. (2019). Biodegradable polymer material based smart stent: Wireless pressure sensor and 3D printed stent. Microelectron. Eng..

[13] Chen W., Habraken T.C.J., Hennink W.E., Kok R.J. (2015). Polymer-Free Drug-Eluting Stents: An Overview of Coating Strategies and Comparison with Polymer-Coated Drug-Eluting Stents. Bioconjug. Chem..

[14] Liu S.J., Chiang F.J., Hsiao C.Y., Kau Y.C., Liu K.S. (2010). Fabrication of balloon-expandable self-lock drug-eluting polycaprolactone stents using micro-injection molding and spray coating techniques. Ann. Biomed. Eng..

[15] Choubey R.K., Pradhan S.K. (2020). Prediction of strength and radial recoil of various stents using FE analysis. Mater. Today Proc..

[16] Saraf A.R., Yadav S.P., Wall J.G., Podbielska H., Wawrzyńska M. (2018). Fundamentals of bare-metal stents. Functionalised Cardiovascular Stents.

[17] Pant S., Bressloff N.W., Limbert G. (2012). Geometry parameterization and multidisciplinary constrained optimization of coronary stents. Biomech. Model. Mechanobiol..

[18] McCormick C., Wall J.G., Podbielska H., Wawrzyńska M. (2018). Overview of cardiovascular stent designs. Functionalised Cardiovascular Stents.

[19] Shen X., Yi H., Ni Z. Effects of Stent Design Parameters on Radial Force of Stent. Proceedings of the 2nd International Conference on Bioinformatics and Biomedical Engineering.

[20] Liu R., Xu S., Luo X., Liu Z. (2020). Theoretical and Numerical Analysis of Mechanical Behaviors of a Metamaterial-Based Shape Memory Polymer Stent. Polymers.

[21] Kumar A., Bhatnagar N. (2020). Finite element simulation and testing of cobalt-chromium stent: A parametric study on radial strength, recoil, foreshortening, and dogboning. Comput. Methods Biomech. Biomed. Eng..

[22] ASTM International F3067-14-Guide for Radial Loading of Balloon Expandable and Self Expanding Vascular Stents. https://www.astm.org/f3067-14.html.

[23] Song K., Bi Y., Zhao H., Wu T., Xu F., Zhao G. (2020). Structural optimization and finite element analysis of poly-l-lactide acid coronary stent with improved radial strength and acute recoil rate. J. Biomed. Mater. Res. Part B Appl. Biomater..

[24] Toong D.W.Y., Ng J.C.K., Huang Y., Wong P.E.H., Leo H.L., Venkatraman S.S., Ang H.Y. (2020). Bioresorbable metals in cardiovascular stents: Material insights and progress. Materialia.

[25] Al-Mangour B., Mongrain R., Yue S. (2013). Coronary Stents Fracture: An Engineering Approach (Review). Mater. Sci. Appl..

[26] Chen C., Xiong Y., Li Z., Chen Y. (2020). Flexibility of biodegradable polymer stents with different strut geometries. Materials.

[27] Karanasiou G.S., Papafaklis M.I., Conway C., Michalis L.K., Tzafriri R., Edelman E.R., Fotiadis D.I. (2017). Stents: Biomechanics, Biomaterials, and Insights from Computational Modeling. Ann. Biomed. Eng..

[28] Xu J., Yang J., Huang N., Uhl C., Zhou Y., Liu Y. (2016). Mechanical response of cardiovascular stents under vascular dynamic bending. Biomed. Eng. Online.

[29] Marrey R.V., Burgermeister R., Grishaber R.B., Ritchie R.O. (2006). Fatigue and life prediction for cobalt-chromium stents: A fracture mechanics analysis. Biomaterials.

[30] Chen F., Ekinci A., Li L., Cheng M., Johnson A.A., Gleadall A., Han X. (2021). How do the printing parameters of fused filament fabrication and structural voids influence the degradation of biodegradable devices?. Acta Biomater..

[31] Luo Q., Liu X., Li Z., Huang C., Zhang W., Meng J., Chang Z., Hua Z. (2014). Degradation Model of Bioabsorbable Cardiovascular Stents. PLoS ONE.

[32] Guerra A.J., Ciurana J. (2018). 3D-printed bioabsordable polycaprolactone stent: The effect of process parameters on its physical features. Mater. Des..

[33] Guerra A.J., Cano P., Rabionet M., Puig T., Ciurana J. (2018). 3D-Printed PCL/PLA Composite Stents: Towards a New Solution to Cardiovascular Problems. Materials.

[34] Zhang Y., Forsyth M., Hinton B., Wallace G.G. (2011). Control of biodegradation of a Mg alloy in simulated body fluid. Aust. Inst. Innov. Mater. Pap..

[35] Bagheri M., Mohammadi M., Steele T.W., Ramezani M. (2016). Nanomaterial coatings applied on stent surfaces. Nanomedicine.

[36] Cockerill I., See C.W., Young M.L., Wang Y., Zhu D. (2021). Designing Better Cardiovascular Stent Materials: A Learning Curve. Adv. Funct. Mater..

[37] Borhani S., Hassanajili S., Ahmadi Tafti S.H., Rabbani S. (2018). Cardiovascular stents: Overview, evolution, and next generation. Prog. Biomater..

[38] Hou R., Wu L., Wang J., Yang Z., Tu Q., Zhang X., Huang N. (2019). Surface-Degradable Drug-Eluting Stent with Anticoagulation, Antiproliferation, and Endothelialization Functions. Biomolecules.

[39] Beshchasna N., Saqib M., Kraskiewicz H., Wasyluk Ł., Kuzmin O., Duta O.C., Ficai D., Ghizdavet Z., Marin A., Ficai A. (2020). Recent Advances in Manufacturing Innovative Stents. Pharmaceutics.

[40] Saleh Y.E., Gepreel M.A., Allam N.K. (2017). Functional Nanoarchitectures For Enhanced Drug Eluting Stents. Sci. Rep..

[41] Ang H.Y., Huang Y.Y., Lim S.T., Wong P., Joner M., Foin N. (2017). Mechanical behavior of polymer-based vs. metallic-based bioresorbable stents. J. Thorac. Dis..

[42] Govindarajan T., Shandas R. (2014). A Survey of Surface Modification Techniques for Next-Generation Shape Memory Polymer Stent Devices. Polymers.

[43] Kereiakes D.J., Onuma Y., Serruys P.W., Stone G.W. (2016). Bioresorbable Vascular Scaffolds for Coronary Revascularization. Circulation.

[44] Komiyama H., Takano M., Hata N., Seino Y., Shimizu W., Mizuno K. (2015). Neoatherosclerosis: Coronary stents seal atherosclerotic lesions but result in making a new problem of atherosclerosis. World J. Cardiol..

[45] Li H., Wang X., Wei Y., Liu T., Gu J., Li Z., Wang M., Zhao D., Qiao A., Liu Y. (2017). Multi-Objective Optimizations of Biodegradable Polymer Stent Structure and Stent Microinjection Molding Process. Polymers.

[46] Konta A.A., García-Piña M., Serrano D.R. (2017). Personalised 3D Printed Medicines: Which Techniques and Polymers Are More Successful?. Bioengineering.

[47] McMahon S., Bertollo N., Cearbhaill E.D.O., Salber J., Pierucci L., Duffy P., Dürig T., Bi V., Wang W. (2018). Bio-resorbable polymer stents: A review of material progress and prospects. Prog. Polym. Sci..

[48] Zia K.M., Noreen A., Zuber M., Tabasum S., Mujahid M. (2016). Recent developments and future prospects on bio-based polyesters derived from renewable resources: A review. Int. J. Biol. Macromol..

[49] Benatti A.C.B., Pattaro A.F., Rodrigues A.A., Xavier M.V., Kaasi A., Barbosa M.I.R., Jardini A.L., Filho R.M., Kharmandayan P., Holban A.M., Grumezescu A.M. (2019). Bioreabsorbable polymers for tissue engineering: PLA, PGA, and their copolymers. Materials for Biomedical Engineering.

[50] Masutani K., Kimura Y., Jiménez A., Peltzer M., Ruseckaite R. (2014). PLA synthesis. From the monomer to the polymer. Poly(lactic acid) Science and Technology: Processing, Properties, Additives and Applications.

[51] Botvin V., Karaseva S., Salikova D., Dusselier M. (2021). Syntheses and chemical transformations of glycolide and lactide as monomers for biodegradable polymers. Polym. Degrad. Stab..

[52] Li S., Vert M., Scott G. (2002). Biodegradation of Aliphatic Polyesters. Degradable Polymers.

[53] Hu Y., Daoud W.A., Cheuk K.K.L., Lin C.S.K. (2016). Newly Developed Techniques on Polycondensation, Ring-Opening Polymerization and Polymer Modification: Focus on Poly(Lactic Acid). Materials.

[54] Ang H.Y., Bulluck H., Wong P., Venkatraman S.S., Huang Y., Foin N. (2017). Bioresorbable stents: Current and upcoming bioresorbable technologies. Int. J. Cardiol..

[55] Singh D., Babbar A., Jain V., Gupta D., Saxena S., Dwibedi V. (2019). Synthesis, characterization, and bioactivity investigation of biomimetic biodegradable PLA scaffold fabricated by fused filament fabrication process. J. Braz. Soc. Mech. Sci. Eng..

[56] Nelson D.L., Cox M.M., Learning M. (2012). Lehninger Principles of Biochemistry.

[57] Sousa A.M., Pinho A.C., Piedade A.P. (2021). Mechanical properties of 3D printed mouthguards: Influence of layer height and device thickness. Mater. Des..

[58] Im S.H., Jung Y., Kim S.H. (2017). Current status and future direction of biodegradable metallic and polymeric vascular scaffolds for next-generation stents. Acta Biomater..

[59] Ang H.Y., Toong D., Chow W.S., Seisilya W., Wu W., Wong P., Venkatraman S.S., Foin N., Huang Y. (2018). Radiopaque Fully Degradable Nanocomposites for Coronary Stents. Sci. Rep..

[60] Pinho A.C., Fonseca A.C., Serra A.C., Santos J.D., Coelho J.F.J. (2016). Peripheral Nerve Regeneration: Current Status and New Strategies Using Polymeric Materials. Adv. Healthc. Mater..

[61] Budak K., Sogut O., Aydemir Sezer U. (2020). A review on synthesis and biomedical applications of polyglycolic acid. J. Polym. Res..

[62] Samantaray P., Little A., Haddleton D., McNally T., Tan B., Sun Z., Huang W., Ji Y. (2020). Poly(glycolic acid) (PGA): A versatile building block expanding high performance and sustainable bioplastic applications. Green Chem..

[63] Gorth D., Webster T.J., Lysaght M., Webster T.J. (2011). Matrices for tissue engineering and regenerative medicine. Biomaterials for Artificial Organs.

[64] Garcia-Garcia H.M., Serruys P.W., Campos C.M., Muramatsu T., Nakatani S., Zhang Y.-J., Onuma Y., Stone G.W. (2014). Assessing Bioresorbable Coronary Devices: Methods and Parameters. JACC Cardiovasc. Imaging.

[65] Erbetta C., Alves R., Resende J., Freitas R., Sousa R. (2012). Synthesis and Characterization of Poly(D,L-Lactide-co-Glycolide) Copolymer. J. Biomater. Nanobiotechnol..

[66] Labet M., Thielemans W. (2009). Synthesis of polycaprolactone: A review. Chem. Soc. Rev..

[67] Bartnikowski M., Dargaville T.R., Ivanovski S., Hutmacher D.W. (2019). Degradation mechanisms of polycaprolactone in the context of chemistry, geometry and environment. Prog. Polym. Sci..

[68] Wang L., Jiao L., Pang S., Yan P., Wang X., Qiu T. (2021). The Development of Design and Manufacture Techniques for Bioresorbable Coronary Artery Stents. Micromachines.

[69] Woodruff M.A., Hutmacher D.W. (2010). The return of a forgotten polymer—Polycaprolactone in the 21st century. Prog. Polym. Sci..

[70] Tenekecioglu E., Farooq V., Bourantas C.V., Silva R.C., Onuma Y., Yılmaz M., Serruys P.W. (2016). Bioresorbable scaffolds: A new paradigm in percutaneous coronary intervention. BMC Cardiovasc. Disord..

[71] Saraf A.R., Sadaiah M. (2017). Photochemical machining of a novel cardiovascular stent. Mater. Manuf. Process..

[72] Raval A., Choubey A., Engineer C., Kothwala D. (2004). Development and assessment of 316LVM cardiovascular stents. Mater. Sci. Eng. A.

[73] Wang C., Zhang L., Fang Y., Sun W. (2021). Design, Characterization, and 3D Printing of Cardiovascular Stents with Zero Poisson’s Ratio in Longitudinal Deformation. Engineering.

[74] Jovic T.H., Combellack E.J., Jessop Z.M., Whitaker I.S. (2020). 3D Bioprinting and the Future of Surgery. Front. Surg..

[75] Giannopoulos A.A., Mitsouras D., Yoo S.-J., Liu P.P., Chatzizisis Y.S., Rybicki F.J. (2016). Applications of 3D printing in cardiovascular diseases. Nat. Rev. Cardiol..

[76] Yeazel T.R., Becker M.L. (2020). Advancing Toward 3D Printing of Bioresorbable Shape Memory Polymer Stents. Biomacromolecules.

[77] Demir A.G., Previtali B. (2017). Additive manufacturing of cardiovascular CoCr stents by selective laser melting. Mater. Des..

[78] Zhao D., Zhou R., Sun J., Li H., Jin Y. (2019). Experimental study of polymeric stent fabrication using homemade 3D printing system. Polym. Eng. Sci..

[79] Piedade A.P. (2019). 4D Printing: The Shape-Morphing in Additive Manufacturing. J. Funct. Biomater..

[80] Pinho A.C., Buga C.S., Piedade A.P. (2020). The chemistry behind 4D printing. Appl. Mater. Today.

[81] Omid S.O., Zahra G., Leila M.K., Ali M., Fateme B. (2020). Self-expanding stents based on shape memory alloys and shape memory polymers. J. Compos. Compd..

[82] Lin C., Zhang L., Liu Y., Liu L., Leng J. (2020). 4D printing of personalized shape memory polymer vascular stents with negative Poisson’s ratio structure: A preliminary study. Sci. China Technol. Sci..

[83] Jia H., Gu S.-Y., Chang K. (2018). 3D printed self-expandable vascular stents from biodegradable shape memory polymer. Adv. Polym. Technol..

[84] Culbreath C.J., Gaerke B., Taylor M.S., McCullen S.D., Mefford O.T. (2020). Effect of infill on resulting mechanical properties of additive manufactured bioresorbable polymers for medical devices. Materialia.

[85] Doshi M., Mahale A., Kumar Singh S., Deshmukh S. (2021). Printing parameters and materials affecting mechanical properties of FDM-3D printed Parts: Perspective and prospects. Mater. Today Proc..

[86] Khan S., Joshi K., Deshmukh S. (2021). A comprehensive review on effect of printing parameters on mechanical properties of FDM printed parts. Mater. Today Proc..

[87] Pinho A.C., Piedade A.P. (2021). Influence of Build Orientation, Geometry and Artificial Saliva Aging on the Mechanical Properties of 3D Printed Poly(ε-caprolactone). Materials.

[88] Chacón J.M., Caminero M.A., García-Plaza E., Núñez P.J. (2017). Additive manufacturing of PLA structures using fused deposition modelling: Effect of process parameters on mechanical properties and their optimal selection. Mater. Des..

[89] Akhoundi B., Behravesh A.H. (2019). Effect of Filling Pattern on the Tensile and Flexural Mechanical Properties of FDM 3D Printed Products. Exp. Mech..

[90] Dudescu C., Racz L. (2017). Effects of Raster Orientation, Infill Rate and Infill Pattern on the Mechanical Properties of 3D Printed Materials. ACTA Univ. Cibiniensis.

[91] Jain S., Fuoco T., Yassin M.A., Mustafa K., Finne-Wistrand A. (2020). Printability and Critical Insight into Polymer Properties during Direct-Extrusion Based 3D Printing of Medical Grade Polylactide and Copolyesters. Biomacromolecules.

[92] Chiulan I., Frone A.N., Brandabur C., Panaitescu D.M. (2018). Recent Advances in 3D Printing of Aliphatic Polyesters. Bioengineering.

[93] Baptista R., Guedes M., Pereira M.F.C., Maurício A., Carrelo H., Cidade T. (2020). On the effect of design and fabrication parameters on mechanical performance of 3D printed PLA scaffolds. Bioprinting.

[94] Jo W., Kwon O.C., Moon M.W. (2018). Investigation of influence of heat treatment on mechanical strength of FDM printed 3D objects. Rapid Prototyp. J..

[95] Kovan V., Altan G., Topal E.S. (2017). Effect of layer thickness and print orientation on strength of 3D printed and adhesively bonded single lap joints. J. Mech. Sci. Technol..

[96] Garzon-Hernandez S., Garcia-Gonzalez D., Jérusalem A., Arias A. (2020). Design of FDM 3D printed polymers: An experimental-modelling methodology for the prediction of mechanical properties. Mater. Des..

[97] Somireddy M., Czekanski A. (2020). Anisotropic material behavior of 3D printed composite structures–Material extrusion additive manufacturing. Mater. Des..

[98] Gonzalez Ausejo J., Rydz J., Musioł M., Sikorska W., Sobota M., Włodarczyk J., Adamus G., Janeczek H., Kwiecień I., Hercog A. (2018). A comparative study of three-dimensional printing directions: The degradation and toxicological profile of a PLA/PHA blend. Polym. Degrad. Stab..

[99] Gao X., Qi S., Kuang X., Su Y., Li J., Wang D. (2021). Fused filament fabrication of polymer materials: A review of interlayer bond. Addit. Manuf..

[100] Ashtankar K.M., Kuthe A.M., Rathour B.S. Effect of Build Orientation on Mechanical Properties of Rapid Prototyping (Fused Deposition Modelling) Made Acrylonitrile Butadiene Styrene (ABS) Parts. Proceedings of the ASME 2013 International Mechanical Engineering Congress and Exposition.

[101] Cherian A.M., Joseph J., Nair M.B., Nair S.V., Maniyal V., Menon D. (2020). Successful Reduction of Neointimal Hyperplasia on Stainless Steel Coronary Stents by Titania Nanotexturing. ACS Omega.

[102] Haryńska A., Carayon I., Kosmela P., Szeliski K., Łapiński M., Pokrywczyńska M., Kucińska-Lipka J., Janik H. (2020). A comprehensive evaluation of flexible FDM/FFF 3D printing filament as a potential material in medical application. Eur. Polym. J..

[103] Algarni M., Ghazali S. (2021). Comparative Study of the Sensitivity of PLA, ABS, PEEK, and PETG’s Mechanical Properties to FDM Printing Process Parameters. Crystals.

[104] Abeykoon C., Sri-Amphorn P., Fernando A. (2020). Optimization of fused deposition modeling parameters for improved PLA and ABS 3D printed structures. Int. J. Lightweight Mater. Manuf..

[105] Lalegani Dezaki M., Ariffin M.K.A.M., Serjouei A., Zolfagharian A., Hatami S., Bodaghi M. (2021). Influence of Infill Patterns Generated by CAD and FDM 3D Printer on Surface Roughness and Tensile Strength Properties. Appl. Sci..

[106] Nam S., Pei E. (2020). The Influence of Shape Changing Behaviors from 4D Printing through Material Extrusion Print Patterns and Infill Densities. Materials.

